# Complete Radiographic Response to Zoledronic Acid Therapy in a Patient With Bony Metastatic Urothelial Carcinoma: A Case Report

**DOI:** 10.4021/wjon244w

**Published:** 2010-11-02

**Authors:** Matthew Kevin Riddle, Albert Craig Lockhart, Stephen M. Sorscher

**Affiliations:** a Washington University Medical School, 4133 Laclede Ave., St. Louis, MO 63108, USA; b Department of Medicine, Oncology Division, Medical Oncology Section, Division of Oncology, Campus Box 8056, Washington University Medical School, 660 South Euclid Avenue, St. Louis, MO 63110, USA

**Keywords:** Zoledronic acid, Bony metastases, Urothelial carcinoma, Radiographic response

## Abstract

Zoledronic acid (ZA) is commonly used for the prevention of skeletal-related events (SREs) in patients with bony metastases. ZA is a bisphosphonate that inhibits osteoclasts, possibly through inhibition of proto-oncogenic Ras activity in osteoclasts by interfering with normal post-translational modifications of Ras. This would suggest that ZA could induce an objective tumor response in metastatic tumors in bone that harbor oncogenic Ras, as the oncogenic Ras in these tumors would require similar post-translational modifications. We recently cared for a patient with biopsy confirmed metastatic urothelial carcinoma who demonstrated a complete radiographic response to ZA. His tumor harbored an oncogenic K-Ras protein, supporting the possibility of interference with Ras activity as the mechanism for the radiographic complete response.

## Introduction

Zoledronic acid (ZA) decreases skeletal-related events (SREs) [1]. It may do so through interfering with proto-oncogenic Ras isoprenylation in osteoclasts [2]. While objective responses to ZA have been noted in renal metastatic disease [3], and ZA treatment has even increased one-year survival rate for patients with metastatic urothelial carcinoma [4], the precise mechanism of action is not known. Herein, we report the first case of a complete radiographic response to ZA in a patient with urothelial carcinoma and suggest that the mechanism of action may be through interference with oncogenic Ras activity.

Oncogenic Ras activity is perhaps the most common oncogenic mechanism involved in human cancer, seen in about one third of human cancers [5]. Based on the described case report, we propose that in the same way that ZA interferes with isoprenylation of osteoclasts, leading to inhibition of osteoclast activity, interference with oncogenic Ras could induce a response in bony metastases containing oncogenic Ras.

## Case Report

JP is a 62 year-old patient who presented in 2007 with a large primary urothelial carcinoma and apparent bony metastases. Biopsies of the primary tumor and the bony metastases confirmed urothelial carcinoma, and molecular studies demonstrated a K-ras mutation in codon 12, a known oncogenic mutation. He received 7 cycles of standard chemotherapy with CDDP (70 mg/m^2^ d1) and Gemcitabine (1000 mg/m^2^ d1, 8 and 15). Following treatment, residual disease was noted on the bone scan, and the patient began ZA therapy (4 mg/1 month). On follow-up scans, the patient was noted to have an objective radiographic response. Subsequent scans, as recently as June 2010, continue to show no evidence of residual bony disease.

## Discussion

To our knowledge, this is the first reported case of a patient with bony metastatic bladder cancer having a complete radiographic response to ZA. Because Ras is a potential downstream target of zoledronic acid, the presence of a K-Ras mutation in the tumor cells may offer a possible explanation for the patient’s robust response. Several independent studies have found the incidence of K-Ras mutation in bladder cancer to be a rare event [6]. We propose the possibility that patients with urothelial and other solid tumors metastatic to bone harboring an oncogenic Ras protein may be candidates for ZA, not only to reduce skeletal related events (SREs) but also to potentially produce an objective response. The biodistribution of ZA is primarily limited to bone; therefore objective tumor responses in other tumor sites, for example liver metastases, would not be anticipated [7]. We plan to test more tumors with bony metastases for oncogenic Ras and to determine if objective responses are demonstrated. ZA may be a 'targeted' therapy in the circumstance of bony metastases harboring an oncogenic Ras mutation.

In conclusion, based on our experience documented in this report, we recommend further research into the mechanism of zoledronic acid’s anti-tumor effect, especially with regard to the role of Ras. Zoledronic acid has already been proven to be an effective therapy in the prevention of skeletal-related events; therefore, many patients with bony metastatic bladder cancer, as well as other tumors, will receive this treatment in the future. A study that prospectively tests patients’ bony metastatic tumors for Ras mutations and then assesses treatment outcomes after ZA therapy could help to determine if tumors harboring oncogenic Ras are especially sensitive to zoledronic acid treatment. Confirmation of this hypothesis could lead to tailored therapies for patients with certain varieties of bony metastatic cancers.
